# Blood cultures taken from patients attending emergency departments in South Africa are an important antibiotic stewardship tool, which directly influences patient management

**DOI:** 10.1186/s12879-015-1127-1

**Published:** 2015-10-06

**Authors:** Tom H. Boyles, Kelly Davis, Thomas Crede, Jacques Malan, Marc Mendelson, Maia Lesosky

**Affiliations:** Division of Infectious Diseases and HIV Medicine, Department of Medicine, Groote Schuur Hospital, University of Cape Town, Observatory, 7925 Cape Town, South Africa; University of Texas Southwestern Medical Center, Dallas, TX USA; Division of Emergency Medicine, Department of Medicine, University of Cape Town, Cape Town, South Africa; Department of Emergency Medicine, Victoria Hospital, Cape Town, South Africa; Department of Medicine, University of Cape Town, Cape Town, South Africa

## Abstract

**Background:**

Febrile illness with suspected blood stream infection (BSI) is a common reason for admission to hospital in Africa and blood cultures are therefore an important investigation. Data on the prevalence and causes of community acquired BSI in Africa are scarce and there are no studies from South Africa. There are no validated clinical prediction rules for use of blood cultures in Africa.

**Methods:**

A prospective observational cohort study of patients attending 2 urban emergency departments in Cape Town, South Africa. The decision to take a blood culture was made by the attending clinician and information available at the time of blood draw was collected. Bottles were weighed to measure volume of blood inoculated.

**Results:**

500 blood culture sets were obtained from 489 patients. 39 (7.8 %) were positive for pathogens and 13 (2.6 %) for contaminants. Significant independent predictors of positive cultures were diastolic blood pressure <60 mmHg, pulse >120 bpm, diabetes and a suspected biliary source of infection, but not HIV infection. Positive results influenced patient management in 36 of 38 (95 %) cases with the organism being resistant to the chosen empiric antibiotic in 9 of 38 (24 %). Taking <8 ml of blood was predictive of a negative culture. The best clinical prediction rule had a negative predictive value (NPV) of 92 % which is unlikely to be high enough to be clinically useful.

**Discussion:**

Blood cultures taken from patients attending emergency departments in a high HIV prevalent city in South Africa are frequently positive and almost always influence patient management. At least 8 ml of blood should be inoculated into each bottle.

**Conclusion:**

Blood cultures should be taken from all patients attending EDs in South Africa suspected of having BSI particularly if diabetic, with hypotension, tachycardia or if biliary sepsis is suspected.

**Electronic supplementary material:**

The online version of this article (doi:10.1186/s12879-015-1127-1) contains supplementary material, which is available to authorized users.

## Background

Febrile illness with suspected blood stream infection (BSI) is a leading reason for admission to hospital [1, 2] and it is important to identify causative organisms with antimicrobial resistance patterns. Where feasible it is therefore common practice to obtain blood cultures during the initial presentation of patients with suspected BSI. A systematic review and meta-analysis of community-acquired BSI in Africa [2] found that 13 % of adult patients presenting with fever had BSI with the commonest causes being *Salmonella typhi*, non-typhoidal *Salmonella* (NTS), *Brucella* species and *Streptococcus pneumoniae* [2]. Human Immunodeficiency Virus (HIV) infection was present in 35 % of those tested and was a significant risk factor for BSI (OR 3.4, *p* < 0.0001). Other significant risk factors for BSI were lethargy, restlessness, oral candidiasis and jaundice.

In high income countries (HICs) BSI has been documented in 1.4 – 8.3 % of blood cultures taken from patients presenting to emergency departments (ED) [3–7], but this influenced patient management in only 0.2-2.8 % of cases where cultures were taken [2–4]. Predominant organisms isolated were *Escherichia coli*, *Streptococci spp.* and *Staphylococcus aureus* [4, 7, 8]. Clinical risk factors for BSI were fever, tachycardia, tachypnoea, chills, hypotension, vomiting and advancing age [3, 7–9].

Optimising the yield from blood cultures is important as negative blood cultures add little to patient management but are costly, cause discomfort to patients, and result in unecessary risk exposures of blood borne viruses to staff. Furthermore, blood that grows one or more contaminant organisms may result in unnecessary antimicrobial treatment and increase the length of hospital stay. A number of studies from HICs with relatively low HIV seroprevalence have developed clinical decision rules to detect which patients presenting to an ED require blood cultures [3, 8]. Such studies are lacking in low- and middle-income countries (LMICs), and in high HIV seroprevalent settings.

There are no published studies of community acquired BSI in South Africa, an upper-middle-income country in Africa [10]. We performed a prospective study of blood cultures taken from patients presenting to 2 EDs in Cape Town, with the primary aim of measuring the incidence of BSI amongst patients suspected of having bacteraemia or fungaemia, to determine the impact of these results on clinical management and clinical risk factors.

## Methods

We performed a prospective observational cohort study of consecutive adult patients (age ≥16 years) presenting to two EDs in Cape Town, South Africa between April and December 2013. The EDs at Groote Schuur Hospital (GSH) and Victoria Hospital (VH) each attend to approximately 40,000 adult patients per year. Both serve urban communities in the City of Cape Town, which had a 19.1 % HIV seroprevalence rate documented in 2010 [11]. Unlike Victoria Hospital, GSH offers a number of specialist services including bone marrow and solid organ transplants.

The decision to perform a blood culture was made independently by the patient’s medical care provider who completed a form with the clinical details available at the time of venesection. Written informed consent was obtained from all patients. The following data was collected for each patient; co-morbidities, the presence of chills, and use of antibiotics in the previous 48 h, temperature, pulse, blood pressure (BP), respiratory rate, oxygen saturation, inspired oxygen concentration and presence of confusion. The suspicion of a focal infection (source) was determined by the attending clinician based on clinical evaluation and was classified into the following categories: lower respiratory tract (pneumonia, bronchitis or acute exacerbation of chronic obstructive pulmonary disease), urinary tract (lower or upper), endocarditis, skin and soft tissue (cellulitis, wet gangrene, necrotising fasciitis, and abscess), biliary tract, gastrointestinal, gynaecological system, meningitis, source of infection unclear and ‘other’.

Hyperthermia was defined as temperature ≥38.3 °C and hypothermia as temperature <36.0 °C. Tachycardia was defined as pulse rate ≥120 beats/min. CRB-65 score was calculated as 1 point for each of; confusion, high respiratory rate ≥30 per minute, low systolic BP < 90 mm Hg or diastolic BP < 60 mmHg, and advanced age ≥65 years [12]. CRB-65 is a validated predictor of mortality from community acquired pneumonia and systemic infections.

A blood culture ‘set’ consisted of a single aerobic bottle. All bottles were weighed after inoculation of blood to determine the volume added. Standard laboratory procedures according to the National Health Laboratory Service (NHLS) in South Africa were used to identify organisms and determine resistance patterns.

The clinical records of all patients with positive blood cultures were reviewed by two infectious disease specialists (TB, MM) to determine whether the organisms represented true pathogens or contaminants. Disagreements were to be adjudicated by a third Infectious Diseases specialist but there were none. Decisions were based on the discretion of the physicians based on the clinical findings in each individual case. Contaminants were common skin commensals (e.g. coagulase-negative staphylococci, “diphtheroids”, micrococcus, Bacillus spp.), which when identified were not in keeping with the clinical features of the case. Clinical records were reviewed to determine the ability of the result to influence patient management. This was assessed by determining if the isolate was grown from another sterile site, and whether the antimicrobials prescribed by the attending clinician were appropriate for the organism.

### Statistical analysis

Frequency (percentage) and median (inter quartile range [IQR]) were calculated for the entire data set as well as for the groups true bacteraemia versus contaminant or no growth. Simple comparisons between groups were done with Fisher’s exact test for discrete variables and Wilcoxon rank sum test for continuous variables. Regression models with a single explanatory variable were carried out to determine the association between potential diagnostic variables and the outcome BSI on the complete data set for the purpose of data exploration and understanding. A number of approaches were explored to find a clinical prediction rule, modelling was done on a randomly selected development sub-sample (50 % of data), including multivariable regression modelling and classification tree analysis. Full statistical methods details available in the online supplement. A clinical prediction rule was considered if the resulting model performance was sufficient (high negative predictive value, NPV) on the validation set, however, no models performed well enough to consider developing a clinical prediction rule. Visual inspection of deviance statistics and partial residual plots were used to check regression assumptions.

Ethical approval for this study was granted by the Faculty of Health Sciences Human Research Ethics Committee, University of Cape Town. Reference 172/2013.

## Results

Four hundred and eighty nine patient encounters were recorded from which 500 blood culture sets were obtained. 410 were from GSH and 90 from VH. A total of 39 (7.8 %) sets were positive with true pathogens and 13 (2.6 %) with contaminants; 4 patients had infection with 2 pathogens and no patient had the same organism isolated from multiple sets. Forty three unique pathogens were identified; 26 were Gram-negative bacteria, 15 Gram-positive bacteria, and 2 were *Cryptococcus neoformans* (Table 1). Two isolates produced extended spectrum beta-lactamase (ESBL) enzymes, and 7 produced inducible beta-lactamase (IBL).

**Table 1:** Organisms identified from 500 blood culture sets taken from 489 patients attending 2 emergency departments in South Africa

Fungal		Gram positive bacterial	3
*Cryptococcus neoformans*	2	*Enterococcus faecalis*	
Gram negative bacteria		*Enterococcus faecium*	3
*Escherichia. coli*	9	*Staphylococcus aureus*	2
*Klebsiella pneumoniae*	5	*Streptococcus pneumoniae*	4
*Klebsiella oxytoca*	1	*Streptococcus constellatus*	1
*Citrobacter sp.*	1	*Streptococcus pyogenes*	1
*Enterobacter sp.*	2	*Gram positive cocci*	
*Morganella sp.*	2	(un-speciated)	1

Table 2 gives the results of the univariate descriptive analysis and the OR (95 % CI) resulting from univariate logisitic regression on the full data set. The summary statistics and results of univariate regression on the derivation and validation subsets (which were utilised in the model building and assessment processes) can be found in the Additional file 1. Median age was 48 years (IQR 31–64) and the median temperature at presentation was 37.4 °C (IQR 36.2-38.4). In terms of comorbidities; 22 % of patients (n = 111) were diabetic and 19 % (n =96) known to be HIV seropositive.

**Table 2:** Frequency (percent) or median (interquartile range) of collected variables in full cohort and by confirmed blood culture results

Characteristic	All data *n* = 500	Blood culture negative *n* = 461	Blood culture positive *n* = 39	OR (95 % CI)
	N (%) or Median (IQR)	N (%) or Median (IQR)	N (%) or Median (IQR)	
Blood volume (mls)	8.89 (6.18, 11.1)	8.83 (6.06, 11)	9.47 (7.5, 11.3)	1.05 (0.97, 1.12)
Weight (kg)	72.6 (69.4, 76.2)	72.6 (69.3, 76.2)	73.1 (70.9, 75.7)	0.99 (0.98, 1)
Age (years)	48 (31, 64)	47 (31, 64)	51 (32, 64.5)	1.01 (0.99, 1.02)
Temperature (C)	37.4 (36.2, 38.4)	37.4 (36.2, 38.4)	37.4 (36.4, 38.5)	1.14 (0.92, 1.45)
Pulse (bpm)	110 (93, 122)	109 (92, 122)	117 (100, 130)	1.02 (1, 1.04)
Respiratory rate (bpm)	22 (18, 28)	22 (18, 28)	24 (20, 29.8)	1.02 (0.98, 1.06)
Oxygen saturations	96 (92, 98)	96 (92, 98)	96 (91, 98)	0.99 (0.94, 1.05)
Inspired oxygen concentration (%)	21 (21, 21)	21 (21, 21)	21 (21, 21)	1.01 (0.99, 1.02)
Chills present	210 (43.8)	190 (43)	20 (52.6)	1.57 (0.83, 3)
Confusion present	142 (29.5)	130 (29.4)	12 (30.8)	1.09 (0.53, 2.11)
CRB 65 score				
0	169 (33.8)	161 (34.9)	8 (20.5)	1.00 (ref)
1	191 (38.2)	173 (37.5)	18 (46.1)	1.69 (0.78, 3.87)
2	101 (20.2)	95 (20.6)	6 (15.4)	1.03 (0.34, 2.85)
3	35 (7)	29 (6.3)	6 (15.4)	3.21 (1.03, 9.32)
4	4 (0.8)	3 (0.6)	1 (2.6)	5.53 (0.26, 47.95)
Diabetic	111 (22.2)	96 (20.8)	15 (38.5)	2.08 (1.04, 4.01)
HIV infected	96 (19.2)	87 (18.9)	9 (23.1)	1.33 (0.6, 2.72)
Hospital				
GSH	409 (82)	374 (81.3)	35 (89.7)	1.00 (ref)
VIC	90 (18)	86 (18.7)	4 (10.3)	0.43 (0.13, 1.1)
Diastolic blood pressure <60 mmHg	138 (28)	118 (26)	20 (51.3)	2.48 (1.3, 4.7)
Systolic blood pressure <90 mmHg	38 (7.7)	30 (6.6)	8 (21.1)	3.26 (1.31, 7.34)
Sex				
Female	263 (52.6)	244 (52.9)	19 (48.7)	1.00 (ref)
Male	237 (47.4)	217 (47.1)	20 (51.3)	1.38 (0.74, 2.63)
Current malignancy present	35 (7)	30 (6.5)	5 (12.8)	2.52 (0.9, 6.09)
Antibiotics in previous 48 h	84 (17.2)	80 (17.8)	4 (10.3)	0.51 (0.15, 1.33)
Source (reduced categories – used in all modelling)				
Others^a^	88 (17.6)	86 (18.7)	2 (5.1)	0.32 (0.05,1.17)
Biliary	19 (3.8)	12 (2.6)	7 (17.9)	9.42 (3.25, 26.83)
LRTI	206 (41.2)	195 (42.3)	11 (28.2)	1.00 (ref)
Meningitis	28 (5.6)	24 (5.2)	4 (10.3)	2.36 (0.63, 7.24)
Unknown	103 (20.6)	93 (20.2)	10 (25.6)	1.59 (0.66, 3.75)
UTI	56 (11.2)	51 (11.1)	5 (12.8)	1.44 (0.45, 4.02)

*P*-value by Fisher’s exact test (discrete variables) or Wilcoxon rank sum test (continuous variables), odd’s ratio (OR) and 95 % confidence interval from univariate logistic regression

*p-value by Wilcoxon rank sum test for continuous variables, Fisher’s exact test for discrete variables except for the reduced source variable. OR (95 % CI) estimated on full data set

^a^Others includes categories: bowel, endocarditis, gynae, other, SSTI

Factors found to be predictive of a positive blood culture on multivariate analysis of the full data set were presence of diabetes OR 2.93 (95 % CI 1.27-6.67) *p* = 0.01, low diastolic BP OR 3.28 (95 % CI 1.24-8.95) *p* = 0.02, high pulse rate OR 2.85 (95 % CI 1.32-6.55) *p* = 0.01, and suspected biliary focus of infection OR 8.72 (95 % CI 2.27-33.1) p = 0.001. Low systolic BP had an increased odds ratio, but did not reach statistical significance; OR 1.49 (95 % CI 0.46-4.60) *p* = 0.49. HIV infection was not associated with BSI.

The continuous covariate, pulse rate, and the discrete covariates, suspected focus of infection, presence of malignancy, low systolic BP, low diastolic BP, high pulse rate, CRB-65 score, high inoculated blood volume were carried forward to the modelling stage based on the univariate p-values from the derivation data set. All continuous variables were scaled at this point to have a mean of zero and standard deviation of one. Regression models with a single explanatory variable for the continuous/discrete pairs were inspected for differences in AIC. The saturated multivariable logistic model included: CRB-65 score (reduced categories – scores of 3 and 4 were pooled), pulse greater than 120 bpm, diastolic BP < 60 mmHg, systolic BP < 90 mmHg, presence of current malignancy, and source (reduced categories). High inoculation blood volume was omitted although significant because it is a variable relating to the patient but one that can be optimised by the clinician. This model was assessed as is, and in a trimmed (by manual elimination of variables with multivariable *p*-value > 0.15) version. The classification tree used only the source variable in the model, which was not instructive. Table 3 describes the results of assessment of diagnostic accuracy of these models and of the classification tree models. No models had a NPV >92 %.

**Table 3:** Performance assessment of multivariable models developed on development subset and validated on validation set (predicting outcomes based on measurements in validation set and comparing with known blood culture result)

Model name	Variables included	Percent correctly classified	Specificity (95 % CI)	NPV (95 % CI)	Sensitivity (95 % CI)	PPV (95 % CI)
Saturated multivariable model (based on univariate *p* < 0.15)	Malignancy, low systolic, low diastolic, high pulse, CRB 65 score, source	91 % (225/248)	0.98 (0.96, 1.00)	0.92 (0.88, 0.95)	0.05 (0.00, 0.25)	0.02 (0.01, 0.72)
Trimmed multivariable model (drop variables with multivariable *p* > 0.15)	Source, CRB 65 score	91 % (225/248)	Same as saturated	Same as saturated	Same as saturated	Same as saturated
Classification tree (only Source used)	All variables (continuous version if available)	90 % (225/250)	0.97 (0.94, 0.99)	0.92 (0.88, 0.95)	0.05 (0.00, 0.25)	0.14 (0.00, 0.45)

### Change in patient management

**Fig. 1:**
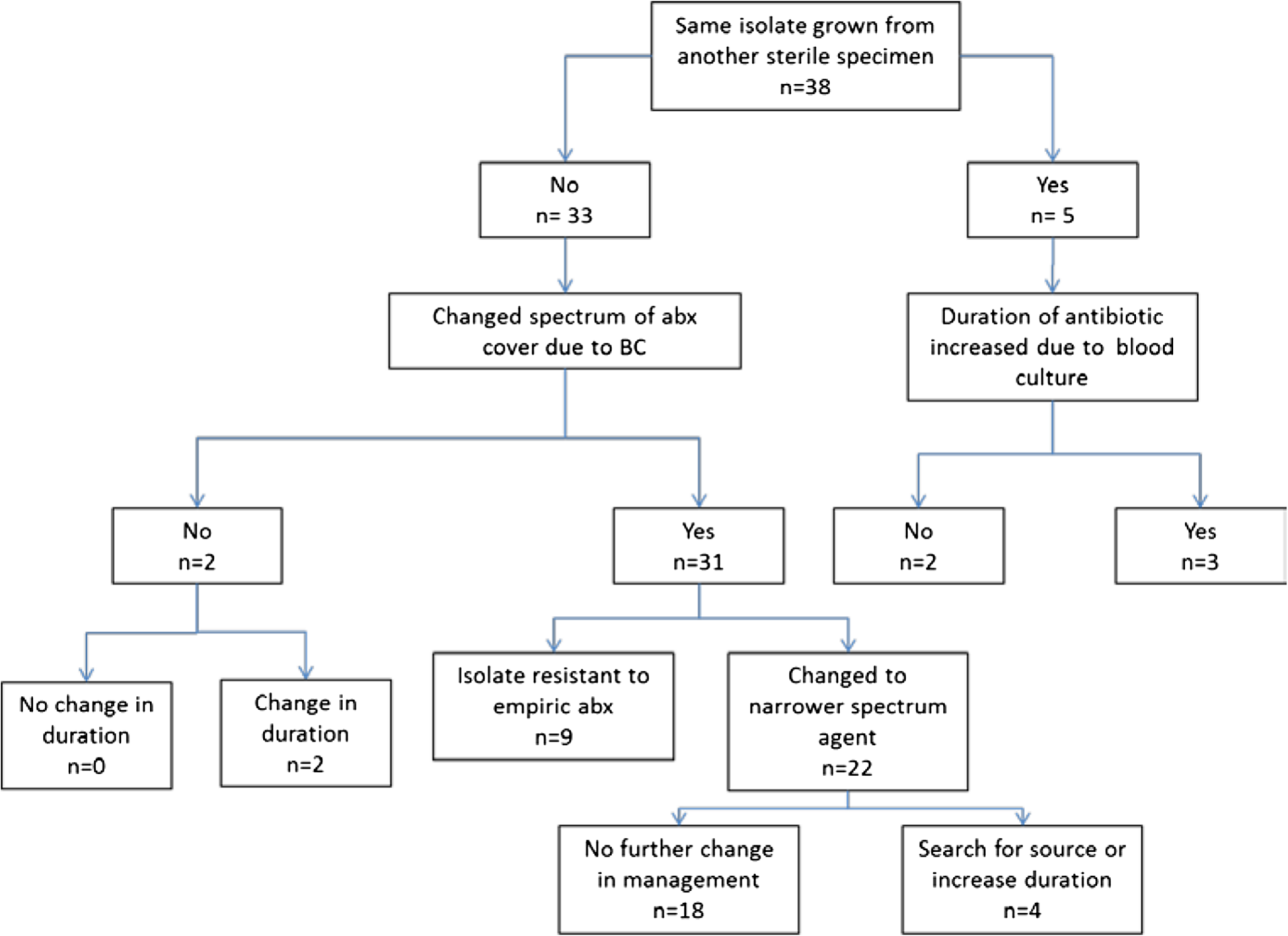
Change in patient management due to 38 positive blood cultures taken from patients attending 2 emergency departments in South Africa

Definitive identification of the organism and antibiotic sensitivities were available for 38 of 39 positive blood culture sets and the influence on patient management is illustrated in Fig. 1. There was no change in management in 2 cases as the causative organism was also cultured from cerebrospinal fluid (CSF). In the remaining 36 of 38 (95 %) cases the result directly influenced patient management. The causative organism was resistant to the empiric therapy chosen by the attending clinician in 9 of 38 (24 %) cases. In 5 of 38 (13 %) cases there was no change in antibiotic spectrum; same organism was cultured from urine (*n* = 3); empiric choice of antibiotic was appropriate for definitive therapy but duration was increased (*n* = 2). In 22 of 38 (58 %) cases the empiric antibiotics where changed to a narrower spectrum agent, in 4 of these the duration of treatment was also significantly extended (2 *Staph. aureus*, 2 Non-typhoidal *Salmonella*).

### Blood volumes

A total of 5.4 % (11/203) of bottles filled with <8 mls of blood were positive compared to 9.6 % (28/291) of bottles filled with >8 mls of blood. The odds of having a positive BC if less than 8 ml of blood were collected was 0.56 times the odds if at least 8 ml of blood is collected OR 0.56 (95 % CI 0.27-1.16).

## Discussion

This is the first prospective study of the utility of blood cultures in ED’s in South Africa and shows that a high proportion of patients (7.8 %) with suspected BSI culture true pathogens. In the vast majority of cases the result directly influences patient management. Notably the organism was resistant to the prescribed empiric antibiotic in a quarter of patients with BSI and the blood culture therefore provided potentially life-saving information. In the remainder the culture results led to either narrowing of the antibiotic spectrum, lengthening of duration of antibiotic therapy or both. Each of these interventions are important components of antimicrobial stewardship. It is therefore important that blood cultures are taken from all patients with suspected BSI attending an ED in South Africa to allow for appropriate antibiotics prescribing, de-escalation and duration of treatment. While patients with negative blood cultures are unlikely to benefit and those with contaminated cultures may be harmed by overuse of antibiotics it is likely that there is an overall benefit to this patient group.

The incidence of BSI in our study is at the upper end of the range with respect to those performed in EDs in HIC’s [3–7] but lower than the average incidence measured in a wide variety of other African settings [2]. Patients in low-income countries (LICs) are more likely to present to hospital with infection than those in HIC’s [13] and as South Africa is defined by the World Bank as an upper-middle income country [10] it is likely that the incidence of infection in general, and BSI in particular, would lie between that of HICs and LICs.

Our results differ from most other studies of BSI in Africa in a number of important ways. Firstly, we did not find HIV infection to be a significant predictor of BSI. This may be related to the low incidence of non-typhoidal *Salmonella* (NTS) infection that we found. HIV is the most important risk factor for NTS in sub-Saharan Africa [14] but we isolated it from only 2 of 96 patients with HIV. There is limited data on the epidemiology of NTS in South Africa but it may be that improved sanitation in urban Cape Town compared to other African cities is an important factor in the low incidence. The incidence of BSI including NTS has also decreased with the widespread introduction of anti-retroviral therapy (ART) in Malawi [15]. ART coverage in Cape Town is high which may partially explain low levels of NTS.

The organisms predominantly causing BSI in Southern Africa are NTS, *Streptococcus pneumoniae* and *Staphylococcus aureus* [2]. In contrast, we found the most common pathogen to be *Escherichia coli*, which is also the most common pathogen in studies from HIC’s. This suggests some similarities between patients from urban Cape Town and HIC’s which might include presence of diabetes and more frequent use of indwelling urinary catheters.

The contamination rate of 2.6 % is lower than in previous ED studies where rates range from 4-6-6 %^3–7^. This may reflect the benefit of dedicated phlebotomy staff, employed approximately 16 h per day, who used sterile technique to draw blood cultures. Blood volume is known to be an independent predictor of blood culture positivity [16]. There was a non-statistically significant trend favouring increased volume of blood in our study. As this is the only variable that can be influenced by the clinician it is important that at least 8 ml of blood is inoculated into all adult blood culture bottles.

A secondary aim of this study was to generate a clinical decision rule with high sensitivity and NPV for blood culture positivity. The highest NPV of any model was 92 % which is equal to the pre-test probability of any culture being negative and means that the model is of no use in discriminating between patients with and without BSI. This failure is surprising given the size of our data set and probably reflects the heterogeneity of patients attending these ED’s. Despite this, our study shows that there are a number of significant predictors of positive blood cultures. Presence of low diastolic BP and tachycardia were predictive of positive culture which is in keeping with the systemic inflammatory response syndrome (SIRS) due to infection. Being diabetic was an independent predictor of positivity that has not been found in other studies [3, 7–9, 17] . The strongest predictor of positivity was a suspected biliary source of infection. The wide confidence intervals limit precise conclusions about the degree of impact of this clinical predictor.

There are a number of limitations to this study. The reference standard for true bacteraemia or fungaemia is the presence of the same organism on 2 consecutive blood cultures. Single cultures were used in this study as it is the standard practice in both ED’s. It is possible that some isolates may have been mis-classified as true pathogens for this reason. However, the clinical records of each positive case were examined by 2 infectious disease specialists to ensure that the organism was compatible with the clinical scenario. Secondly, sample collection was determined by the attending clinicians rather than by strict criteria and were not taken from consecutive patients as some clinicians may have taken blood cultures without filling out the necessary data collection form. It is not known whether this introduced a patient selection bias. Clinicians only chose patients for blood cultures if they had a suspicion of BSI which is dependent on clinical skill and experience. It is not possible to determine the rate of BSI among patients who were not considered to be at risk of BSI by clinicians. Strengths of the study are that it was prospective and had a pragmatic design meaning that only parameters that would be available to clinicians at the time of blood draw under standard working conditions were incorporated into the model. We chose 2 different EDs to include a broad mix of patients, more likely to be representative of patients presenting to urban centres in South Africa.

## Conclusions

In conclusion, correctly taken blood cultures (at least 8 ml of blood for each culture) are an important antibiotic stewardship tool in patients attending EDs in South Africa. It is not possible to provide a rule for patients who do not require blood cultures and they should therefore be performed on all patients with suspected BSI particularly if there is a SIRS response or the patient is diabetic.

## Additional file

Additional file 1:
**Online supplement.** (DOCX 15 kb)

## References

[1] Angus DC, Linde-Zwirble WT, Lidicker J, Clermont G, Carcillo J, Pinsky MR (2001). Epidemiology of severe sepsis in the united states: Analysis of incidence, outcome, and associated costs of care. Crit Care Med.

[2] Reddy EA, Shaw AV, Crump JA (2010). Community-acquired bloodstream infections in africa: A systematic review and meta-analysis. The Lancet Infectious Diseases.

[3] Shapiro NI, Wolfe RE, Wright SB, Moore R, Bates DW (2008). Who needs a blood culture? A prospectively derived and validated prediction rule. J Emerg Med.

[4] Howie N, Gerstenmaier JF, Munro PT (2007). Do peripheral blood cultures taken in the emergency department influence clinical management?. Emerg Med J.

[5] Kelly AM (1998). Clinical impact of blood cultures taken in the emergency department. J Accid Emerg Med.

[6] Mountain D, Bailey PM, O'Brien D, Jelinek GA (2006). Blood cultures ordered in the adult emergency department are rarely useful. Eur J Emerg Med.

[7] Stalnikowicz R, Block C (2001). The yield of blood cultures in a department of emergency medicine. Eur J Emerg Med.

[8] Su CP, Chen TH, Chen SY (2011). Predictive model for bacteremia in adult patients with blood cultures performed at the emergency department: A preliminary report. J Microbiol Immunol Infect.

[9] Coburn B, Morris AM, Tomlinson G, Detsky AS (2012). Does this adult patient with suspected bacteremia require blood cultures?. JAMA.

[10] World Bank. Country and lending groups 2015. http://data.worldbank.org/about/country-and-lending-groups#Upper_middle_income. Accessed 01/23, 2015.

[11] National Department of Health, South Africa. The national antenatal sentinel HIV and syphilis prevalence survey. http://www.gov.za/sites/www.gov.za/files/hiv_aids_survey_a_0.pdf. Updated 2010. Accessed 11/11, 2014.

[12] Bauer TT, Ewig S, Marre R, Suttorp N, Welte T, CAPNETZ Study Group (2006). CRB-65 predicts death from community-acquired pneumonia. J Intern Med.

[13] GBD 2013 Mortality and Causes of Death Collaborators. Global, regional, and national age-sex specific all-cause and cause-specific mortality for 240 causes of death, 1990–2013: A systematic analysis for the global burden of disease study 2013. Lancet. 2014;385(9963):p117-171. 10.1016/S0140-6736(14)61682-2 PMC4340604 25530442

[14] Feasey NA, Dougan G, Kingsley RA, Heyderman RS, Gordon MA (2012). Invasive non-typhoidal salmonella disease: An emerging and neglected tropical disease in africa. Lancet.

[15] Feasey NA, Houston A, Mukaka M (2014). A reduction in adult blood stream infection and case fatality at a large african hospital following antiretroviral therapy roll-out. PLoS One.

[16] Gonsalves WI, Cornish N, Moore M, Chen A, Varman M (2009). Effects of volume and site of blood draw on blood culture results. J Clin Microbiol.

[17] Chase M, Klasco RS, Joyce NR, Donnino MW, Wolfe RE, Shapiro NI (2012). Predictors of bacteremia in emergency department patients with suspected infection. Am J Emerg Med.

