# Supplementary material for: Blood cultures taken from patients attending emergency departments in South Africa are an important antibiotic stewardship tool, which directly influences patient management
Source: BMC Infect Dis. 2015 Oct 6;15:410. doi: 10.1186/s12879-015-1127-1 (PMC4594638; doi:10.1186/s12879-015-1127-1)
Supplement: Additional file 1: — Online supplement. (DOCX 15 kb) [file 12879_2015_1127_MOESM1_ESM.docx]

Online supplement

Detailed statistical methods

The data set was split into development and validation subsets, each with 50% of the data. The development data set was used for all model building. Logistic regression with a single explanatory variable was carried out for each candidate variable and those with p < 0.15 were carried forward to multivariable regression models. In the event that both continuous and discrete versions of the variable were candidates, alternate models were inspected and unless the Akaike information criterion (AIC) difference between the models was large (>>2), the discrete version of the variable was retained. The full multivariable model was inspected and trimmed, dropping variables with a multivariable p-value > 0.15. Deviance and partial residual plots were inspected to assess model diagnostics. The saturated and trimmed multivariable models were applied to the validation set and sensitivity, specificity, and positive and negative predictive values estimated. Classification tree analysis was also attempted, using continuous versions of variables as inputs, however the resulting tree only retained the source variable making it non-informative.
